# Blood pressure trajectories in extreme preterm infants with and without bronchopulmonary dysplasia: a longitudinal cohort study

**DOI:** 10.1186/s12887-026-06777-8

**Published:** 2026-03-25

**Authors:** Mohammad Shawaqfeh, Mohamed Sayed Alshaar, Abdulrahman Debs, Sofyan Lafi, Khuloud Mohamed, Seba Abounahia, Mohammad A. A. Bayoumi, Ashraf Gad

**Affiliations:** 1 Neonatal Intensive Care Unit, Women’s Wellness and Research Center, Hamad Medical Corporation, P.O. Box: 3050, Doha, Qatar; 2 Department of Medical Education, Sidra Medicine, Doha, Qatar; 3 College of Medicine, Bahcesehir University, Istanbul, Turkey; 4 Weill Cornell-Qatar, Doha, Qatar

**Keywords:** Bronchopulmonary dysplasia, Extremely preterm infants, Blood pressure, Neonatal hypertension, Hemodynamics, Postmenstrual age, Non-invasive blood pressure monitoring, Neonatal intensive care unit, Pulmonary morbidity, Longitudinal cohort study

## Abstract

**Objective:**

To compare longitudinal blood pressure (BP) patterns in extremely preterm (EP) infants with and without bronchopulmonary dysplasia (BPD) and examine associations with clinical characteristics.

**Methods:**

This retrospective cohort study analysed weekly non-invasive systolic, diastolic, and mean BP measurements from 28 weeks postmenstrual age (PMA) to term infants cared for at the Women’s Wellness and Research Center, Doha. BP trajectories were compared longitudinally, with additional analysis by BPD severity.

**Results:**

Among 201 infants, 95 (47%) developed BPD. These infants were born at lower gestational age and birth weight and had greater exposure to respiratory therapies, higher rates of patent ductus arteriosus, longer hospitalisation, and more frequent cardiovascular support.

BP trajectories differed between groups. At 28–29 weeks PMA, systolic, diastolic, and mean BPs were slightly lower in infants with BPD. From approximately 32–36 weeks PMA, BP values converged, and later PMAs showed higher systolic and mean pressures in the BPD group. The proportion of systolic BP values above the 95th percentile was generally higher among infants with BPD, although differences were not consistently significant.

In multivariable regression, BPD was not independently associated with hypertensive BP episodes. Male sex was associated with lower odds of hypertensive systolic, diastolic, and mean BP episodes, while higher birth weight was associated with fewer systolic hypertensive episodes.

**Conclusion:**

EP infants with BPD demonstrate distinct BP trajectories, characterised by early relative hypotension followed by higher systolic pressures later in hospitalisation. However, BPD was not independently associated with hypertensive BP episodes. Sex and physiologic maturity appear to be stronger determinants of BP variability, supporting the importance of longitudinal BP monitoring in EP infants.

## Introduction

Extreme preterm (EP) infants are highly susceptible to cardiovascular and hemodynamic disturbances [1]. These vulnerabilities are compounded by many factors such as intrauterine growth restriction, the need for prolonged respiratory support and exposure to interventions that may further affect systemic and pulmonary circulation [2, 3]. Cardiac remodelling and altered vascular reactivity in this population contribute to complex circulatory patterns during the neonatal period [4].

Bronchopulmonary dysplasia (BPD) is known to be one of the most prematurity-associated morbidities, accounting for 29 to 47% in surviving EP infants [5, 6]. In addition to chronic respiratory complications, preterm infants who survive with BPD are at increased risk for cardiovascular consequences, including pulmonary hypertension (PH), cardiac dysfunction, reduced exercise capacity, and other lifelong complications [7, 8].

One potential but relatively underexplored issue in extremely preterm infants with BPD is whether they exhibit different blood pressure (BP) patterns and a possible increased tendency toward systemic hypertension [9]. The mechanisms underlying this possible association remain uncertain and are likely multifactorial. Infants with BPD may experience chronic hypoxemia and intermittent hypercarbia, which can stimulate peripheral chemoreceptors and increase sympathetic activity, potentially raising systemic vascular resistance [10]. Persistent inflammation and oxidative stress may also affect endothelial function and activate the renin–angiotensin–aldosterone system (RAAS), mechanisms that could influence BP regulation [11]. In addition, extreme prematurity itself may predispose to later hypertension due to disrupted nephrogenesis and reduced nephron number [12]. NICU interventions such as corticosteroids, inotropic support, and oxygen therapy may also affect BP in this population [10, 13].

Although reference ranges for neonatal BP have been proposed based on gestational and postnatal age, few studies have systematically evaluated the incidence of systemic hypertension in infants with BPD compared to those without [14]. In a recent retrospective study of 26 preterm infants with BPD, investigators observed significantly elevated systolic BP during the first three months of life. Notably, 42% of these infants had hypertensive BP readings sustained for at least three days within the initial 90 days [9].

BP trajectories may also provide a simple, noninvasive marker of evolving cardiopulmonary vascular load, complementing echocardiographic assessment, and may aid screening and follow-up planning for this high-risk population. Accordingly, this longitudinal observational cohort study describes and compares cuff-measured BP trajectories from 28 to 40 weeks’ postmenstrual age (PMA) in EP infants with and without BPD, to define BPD-associated patterns, identify periods of increased risk, and generate data to inform future studies.

## Methods

### Study population and sampling strategy

This retrospective cohort study was conducted at the Women’s Wellness and Research Center (WWRC), Hamad Medical Corporation (HMC) in Doha, Qatar. Approval for the study was obtained from the Institutional Review Board (IRB) and the ethical committee of the MRC at Hamad Medical Corporation (HMC), Qatar, under protocol number (MRC-01-22-236). Consent to participate was waived by the IRB at HMC in accordance with national regulations, as the study is retrospective and observational. All methods were performed in accordance with the relevant guidelines and regulations.

The study included all preterm infants who were born less than 28 weeks’ gestation and admitted to the WWRC Hospital from January 2018 until December 2019. We identified 230 infants. Of them, 201 were included in the analysis. Exclusion criteria were infants who died before 12 h (*n* = 7), infants who died before 36 weeks PMA (*n* = 21), and infants with congenital anomalies or heart or renal disease (*n* = 2).

In the study, we compared weekly-averaged noninvasive readings of systolic, diastolic, and mean BP in EP infants with and without BPD from the corrected age of 28 weeks until discharge from the neonatal intensive care unit (NICU). BP readings that were excluded if recorded via invasive methods (Umbilical or invasive arterial catheter), during episodes of culture-proven or clinical sepsis, ventilator-associated pneumonia, shock or other significant infection and septic shock. Additionally, those recorded while the infant is on a medication that can alter the BP, including systemic steroids and inotropes. We did not exclude those on a diuretic course. BP was measured non-invasively using an oscillometric method (UniMed neonatal cuff system) according to manufacturer specifications. Appropriate cuff size was selected based on limb circumference: size 1 (3–6 cm), size 2 (4–8 cm), size 3 (6–11 cm), size 4 (7–13 cm), and size 5 (8–15 cm). All measurements were obtained using standardised Philips bedside monitors and were automatically recorded in the electronic medical record. In total, 6,848 weekly-averaged blood pressure measurements (systolic, diastolic, and mean) were included in the analysis.

BPD severity was classified according to the Jensen criteria, which stratify infants based on the mode of respiratory support at 36 weeks PMA into three grades: Grade 1 (nasal cannula ≤ 2 L/min), Grade 2 (non-invasive support or nasal cannula > 2 L/min), and Grade 3 (invasive mechanical ventilation) [15].

### Study variables

Data sets included basic demographic variables for all newborns, including gestational age (GA), gender, birth weight (BW), mode of delivery, and Apgar scores. Non-invasive BP readings were collected over a one-week gestation for each newborn, then averaged and rounded to two decimal places, excluding outliers. Other variables that might affect BP were collected, including episodes of sepsis, inotropic support, use of postnatal steroids or diuretics, duration of umbilical artery catheterisation, cardiac echocardiographic findings, and renal ultrasound results, if performed.

### Statistical analysis

Descriptive analyses were conducted to evaluate patient characteristics and clinical variables. Continuous variables were summarised as mean and standard deviation (SD) for parametric data, and as median and interquartile range (IQR) for non-parametric data. Comparisons of continuous variables were made using Student’s t-test for parametric distributions or the Mann-Whitney U test for nonparametric distributions, as appropriate. Categorical variables were presented as frequencies and percentages and compared using chi-square or Fisher’s exact tests. Weekly systolic, diastolic, and mean blood pressure values from 28 to 40 weeks’ PMA were compared between infants with bronchopulmonary dysplasia (BPD) and those without BPD to assess differences in longitudinal blood pressure patterns. Multivariable logistic regression analyses were performed to identify independent predictors of hypertensive systolic, diastolic, and mean BP episodes, including clinically relevant perinatal and treatment variables. All p-values were two-tailed, and values below 0.05 were considered statistically significant. Statistical analyses were conducted using SPSS software version 30 (IBM Corp., Armonk, NY).

## Results

The study involved 201 preterm infants who met the inclusion criteria; among them, 95 infants had BPD, and 106 were labelled as non-BPD. The baseline characteristics of infants with and without BPD are summarised in Table 1. Infants who developed BPD were born at a significantly lower GA compared with those without BPD (25.2 ± 1.3 vs. 25.9 ± 1.1 weeks, *p* = 0.006) and had lower BW (795 ± 143 g vs. 914 ± 194 g, *p* = 0.011). Male sex was more prevalent among infants with BPD (65.3% vs. 48.1%; OR 2.03, 95% CI 1.15–3.58, *p* = 0.014).

There were no significant differences between groups in rates of small for gestational age, multiple gestation, antenatal steroid exposure, maternal hypertension, chorioamnionitis, or premature rupture of membranes. Infants who developed BPD were more likely to have lower Apgar scores at 1 min (80.0% vs. 59.4%; OR 2.73, 95% CI 1.45–5.15, *p* = 0.002), although differences at 5 min did not reach statistical significance.

**Table 1:** Baseline Perinatal Characteristics of Infants with and without Bronchopulmonary Dysplasia

Variable	No BPD (*n* = 106)	BPD (*n* = 95)	Odds Ratio/Mean Difference (95% CI)	*p* value
Gestational age (weeks)	25.94 ± 1.07	25.21 ± 1.28	0.73 (0.20, 1.27)	0.006
Birth weight (g)	914 ± 194	795 ± 143	119 (28, 211)	0.011
Male sex	51 (48.1%)	62 (65.3%)	2.03 (1.15, 3.58)	0.014
Small for gestational age	5 (4.7%)	5 (5.3%)	1.12 (0.32, 4.00)	0.859
Multiple gestation	38 (35.8%)	32 (33.7%)	0.91 (0.51, 1.63)	0.748
Vaginal delivery	42 (39.6%)	40 (42.1%)	1.11 (0.63, 1.95)	0.721
Antenatal steroids	92 (86.8%)	79 (83.2%)	0.75 (0.34, 1.64)	0.470
Maternal gestational diabetes	16 (15.1%)	15 (15.8%)	1.05 (0.49, 2.27)	0.892
Maternal hypertension	10 (9.4%)	11 (11.6%)	1.26 (0.51, 3.11)	0.620
Chorioamnionitis	17 (16.0%)	10 (10.5%)	0.62 (0.27, 1.42)	0.253
PROM	27 (25.5%)	24 (25.3%)	0.99 (0.52, 1.87)	0.973
Low Apgar (< 7 at 1 min)	63 (59.4%)	76 (80.0%)	2.73 (1.45, 5.15)	0.002
Low Apgar (< 7 at 5 min)	17 (16.0%)	22 (23.2%)	1.58 (0.78, 3.19)	0.203

Values are presented as mean ± SD or number (%)

Odds ratios (ORs) with 95% confidence intervals (CIs) are shown for categorical variables

*BPD* Bronchopulmonary dysplasia, *GA* Gestational age, *PROM* Premature rupture of membranes

Infants who developed BPD demonstrated significantly greater illness severity compared with those without BPD (Table 2). Infants who developed BPD (*n* = 95) had significantly greater exposure to respiratory support and postnatal steroid therapy compared with infants without BPD (*n* = 106). Surfactant administration was more frequent in the BPD group (94.7% vs. 75.5%; OR 5.85, 95% CI 2.15–15.96; *p* < 0.001). Duration of mechanical ventilation was significantly longer among infants with BPD (median 11 days [IQR 2–35]) compared with those without BPD (median 1 day [IQR 0–4]; *p* < 0.001).

Exposure to systemic postnatal steroids was strongly associated with BPD. Infants with BPD were more likely to receive both systemic and inhaled steroids (38.9% vs. 2.8%; OR 21.90; *p* < 0.001), and (22.3% vs. 1.9%; OR 14.05; *p* < 0.001), respectively. However, the timing of steroid initiation (day of life or PMA) did not differ between groups.

Umbilical arterial catheter use was more frequent (68.4% vs. 45.3%; OR 2.62; *p* < 0.001), and catheter duration was longer (7.4 ± 3.4 vs. 5.2 ± 2.5 days; *p* = 0.004). In contrast, late-onset sepsis after two weeks of life did not differ significantly between groups. Infants with BPD also had a later PMA at discharge (41.6 weeks [IQR 39.4–46.4] vs. 36.3 weeks [IQR 35.0–38.2]; *p* < 0.001) and a longer hospital stay (119 days [IQR 95–148.5] vs. 71.5 days [IQR 61.8–90.0]; *p* < 0.001).

Mortality was higher in the BPD group both overall and before 36 weeks PMA, but these differences did not reach statistical significance.

**Table 2:** Clinical characteristics and in-hospital outcomes in infants with and without bronchopulmonary dysplasia

Variable	No BPD (*n* = 106)	BPD (*n* = 95)	Odds Ratio/Mean Difference (95% CI)	*p* value
Surfactant	80/106 (75.5%)	90/95 (94.7%)	5.85 (2.15, 15.96)	< 0.001
Ventilation duration (days)	1 (0, 4)	11 (2, 35)	–	< 0.001
Systemic PNS (DART)	3/106 (2.8%)	37/95 (38.9%)	21.90 (6.45, 74.17)	< 0.001
Systemic PNS start day of life (days)	33 ± 26.9 (3)	32.2 ± 16.2 (37)	0.76 (–19.63, 21.15)	0.940
Systemic PNS start PMA (weeks)	29.3 ± 4.1 (3)	29.9 ± 2.7 (37)	–0.57 (–3.94, 2.83)	0.740
Inhaled postnatal steroids	2/106 (1.9%)	20/94 (22.3%)	14.05 (3.19, 61.97)	< 0.001
Inhaled PNS start (day of life)	61.5 (50, – ^a^) (2)	56 (45–81.5) (20)	—	0.819
Inhaled PNS start PMA (weeks)	34.2 (33.1, –^a^) (2)	32.6 (30.6–37.8) (20)	—	0.512
UAC	48/95 (45.3%)	65/95 (68.4%)	2.62 (1.47, 4.66)	< 0.001
UAC duration (days)	5.2 ± 2.5 (48)	7.4 ± 3.4 (65)	–2.69 (–4.49, − 0.89)	0.004
Sepsis after 2 weeks of life	13/106 (12.3%)	13/95 (13.7%)	1.13 (0.50, 2.59)	0.765
Culture-proven sepsis (day of life)	33.3 ± 20.2 (13)	33.5 ± 28.0 (2)	–0.15 (–22.4, 22.16)	0.026
Culture-proven sepsis PMA (weeks)	30.9 ± 2.8 (13)	30.2 ± 4.1 (2)	0.75 (0.08, 7.75)	0.048
Discharge PMA (weeks)	36.3 (35.0–38.2)	41.6 (39.4–46.4)	—	< 0.001
Hospital stay (days)	71.5 (61.8–90.0)	119 (95–148.5)	—	< 0.001
Death, any time	2/106 (1.9%)	6/95 (6.3%)	3.51 (0.69, 17.81)	0.109
Death < 36 weeks PMA	1/106 (0.9%)	5/95 (5.3%)	5.83 (0.67, 50.86)	0.072

Values are presented as mean ± SD (*n*, if missing values) or *n* (%), or median (IQR) (*n*, if subgroup or missing values)

Odds ratios (OR) or mean differences are shown with 95% confidence intervals

*BPD* Bronchopulmonary dysplasia, *PMA* Postmenstrual age, *UAC* Umbilical arterial catheter, *PNS* Postnatal steroids (DART, dexamethasone: a randomised trial), *CI* Confidence interval, *OR* Odds ratio

^a﻿^The IQR cannot be calculated properly because the sample size for those variables in the No-BPD group is extremely small

Table 3 summarises the cardiovascular characteristics and treatment exposures of infants with and without BPD. Vasopressor use was more frequent in the BPD group (9.5% vs. 2.8%; OR 3.59, 95% CI 0.94–13.69, *p* = 0.047), although the timing of vasopressor use by day of life or PMA did not differ significantly between groups. Echocardiographic evaluation of PDA by 36 weeks’ PMA was performed in 68.4% of infants with BPD compared with 32.1% of those without BPD (OR 4.59, 95% CI 2.53–8.32, *p* < 0.001).

Infants with BPD were also more likely to receive medical treatment for PDA (68.4% vs. 45.5%; OR 2.59, 95% CI 1.45–4.64; *p* = 0.001) and were more likely to undergo device closure (11.1% vs. 2.2%; OR 5.56, 95% CI 1.18–26.15; *p* = 0.016). The presence of PDA at 36 weeks PMA and the prevalence of pulmonary hypertension and left ventricular hypertrophy were low and did not differ significantly between groups.

Exposure to diuretic therapy was markedly higher among infants with BPD, with 57.9% receiving at least one diuretic compared with 11.3% of infants without BPD (OR 10.77, 95% CI 5.21–22.26, *p* < 0.001). Similarly, furosemide use alone was significantly more common in the BPD group (15.8% vs. 2.8%; OR 6.44, 95% CI 1.80–23.00, *p* = 0.001). Renal ultrasound evaluation was also more frequently performed in infants with BPD (33.7% vs. 13.2%; OR 3.34, 95% CI 1.65–6.76, *p* < 0.001), although the prevalence of abnormal findings did not differ significantly between groups.

**Table 3:** Cardiovascular and Hemodynamic Outcomes in Infants with and without Bronchopulmonary Dysplasia

Variable	No BPD (*n* = 106)	BPD (*n* = 95)	Odds Ratio/Mean Difference (95% CI)	*p* value
Vasopressor therapy	3 (2.8)	9 (9.5)	3.59 (0.94, 13.69)	0.047
Vasopressors use day of life (days)	73.5 (46.0, – ^a^) (2)	57.5 (42, 169.8) (6)	—	1.000
Vasopressors use PMA (weeks)	35.5 (32.6, – ^a^) (2)	33.2 (29.7, 48.8) (6)	—	1.000
Left ventricular hypertrophy	1 (1.0)	2 (2.1)	2.22 (0.20, 24.83)	0.508
Echocardiogram at 36 weeks PMA	34 (32.1)	65 (68.4)	4.59 (2.53, 8.32)	< 0.001
PDA present at 36 weeks PMA	1 (0.9)	3 (3.2)	3.42 (0.35, 33.49)	0.262
PDA medically treated	46 (45.5)	65 (68.4)	2.59 (1.45, 4.64)	0.001
PDA Device closure	2 (2.2%)	10 (11.1%)	5.56 (1.18, 26.15)	0.016
Pulmonary hypertension at 36 weeks PMA	1 (1.0%)	2 (2.2%)	2.29 (0.20, 25.71)	0.489
Anti-hypertensive therapy (not diuretics)	0 (0.0)	2 (2.1)	—	0.133
Any diuretic exposure for BPD	12 (11.3%)	55 (57.9%)	10.77 (5.21, 22.26)	< 0.001
Diuretic start day of life (days)	43.6 ± 15.1 (11)	47.8 ± 23.8 (55)	–4.11 (–19.14, 10.85)	0.584
Diuretic start PMA (weeks)	31.7 ± 2.1 (11)	32.1 ± 2.9 (55)	–4.20 (–2.84, 2.00)	0.730
Furosemide use for BPD	3 (2.8%)	15 (15.8%)	6.44 (1.80, 23.00)	0.001
Renal ultrasound performed	14 (13.2%)	32 (33.7%)	3.34 (1.65, 6.76)	< 0.001
Abnormal renal ultrasound	3 (21.4%)	9 (28.1%)	1.44 (0.32, 6.37)	0.634

Values are presented as mean ± SD (*n*, if missing values) or *n* (%), or median (IQR) (*n*, if subgroup or missing values). Odds ratios (ORs) with 95% confidence intervals (CIs) compare infants with bronchopulmonary dysplasia (BPD) to those without BPD. PMA, postmenstrual age; PDA, patent ductus arteriosus

^a﻿^The IQR cannot be calculated properly because the sample size for those variables in the No-BPD group is extremely small

Table 4 summarises longitudinal blood pressure measurements for the entire cohort across PMAs (28–40 weeks). Across all infants, systolic, diastolic, and mean arterial pressures increased progressively with advancing PMA, reflecting normal postnatal cardiovascular maturation. Mean systolic BP increased from approximately 65 mmHg at 28 weeks to over 80 mmHg by 40 weeks, with parallel upward trends observed for diastolic and mean arterial pressures.

**Table 4:** Postmenstrual age-specific blood pressure reference values in extremely preterm infants

PMA(weeks)	Patients (n)	Systolic Mean	Systolic 5^th^	Systolic 95^th^	Diastolic Mean	Diastolic 5^th^	Diastolic 95^th^	MAP Mean	MAP 5^th^	MAP 95^th^
28	197	65	54	80	38	29	48	47	38	58
29	201	67	56	81	39	31	48	49	40	58
30	199	69	59	80	40	33	49	50	43	59
31	199	70	61	81	41	33	50	50	43	60
32	193	70	61	82	41	34	50	51	44	59
33	194	71	62	82	41	34	51	51	44	60
34	190	73	63	83	42	36	49	52	45	60
35	177	75	65	84	43	37	52	54	46	62
36	149	76	66	87	45	37	53	55	47	64
37	119	77	68	89	45	38	54	56	48	65
38	99	78	68	91	46	38	58	57	49	69
39	94	80	71	91	47	39	55	58	50	67
40	77	81	72	93	47	40	56	59	51	69

Values are presented as mean and 5th–95th percentile ranges unless otherwise stated. Blood pressure (BP) measurements were obtained using non-invasive oscillometric methods and summarised by postmenstrual age (PMA)

*PMA* Postmenstrual age, *mmHg*, Millimetres of mercury

**Fig. 1:**
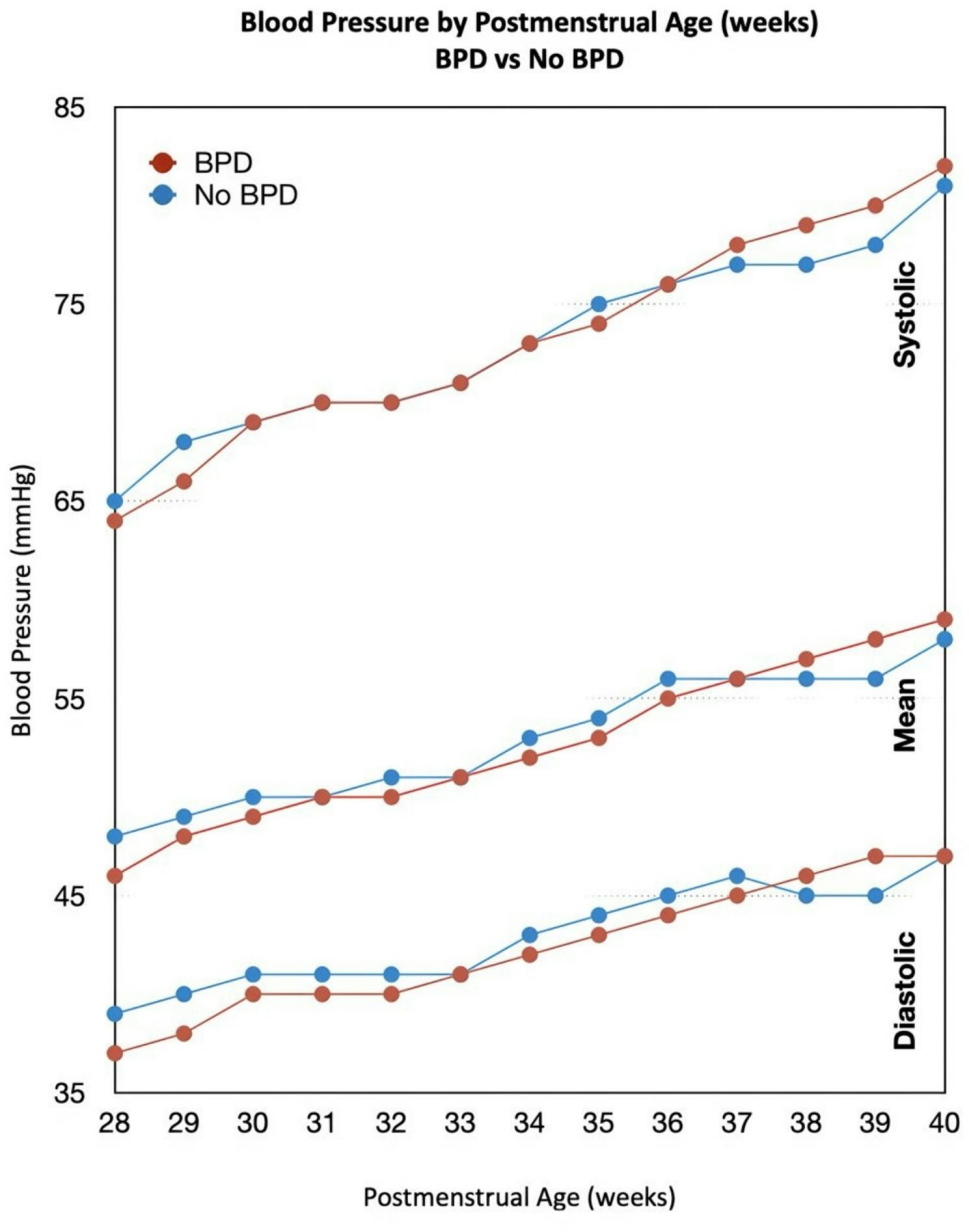
Blood pressure trajectories by bronchopulmonary dysplasia status

Table 5, 6, and 7 stratify BP trajectories by BPD status. Infants who developed BPD consistently exhibited higher BP values compared with those without BPD across most PMAs. This difference was most pronounced for systolic BP (Table 5), particularly from 32 weeks’ PMA onward, where infants with BPD showed higher mean and upper percentile values compared with their non-BPD counterparts (Figure 1). Differences in diastolic and mean arterial BPs (Tables 6 & 7) were more modest but followed a similar directional trend.

**Table 5:** Weekly systolic blood pressure by postmenstrual age (mmhg) in infants with and without bronchopulmonary dysplasia

PMA (weeks)	No BPDSystolic BP (mmHg)			BPDSystolic BP (mmHg)		
	GAMean	5^th^	95^th^	Mean	5^th^	95^th^
28	65	58	79	64	49	81
29	68	60	79	66	50	81
30	69	60	78	69	58	81
31	70	61	80	70	61	83
32	70	61	81	70	61	83
33	71	62	82	71	62	83
34	73	62	82	73	64	84
35	75	66	84	74	65	85
36	76	66	87	76	66	87
37	77	65	88	78	68	90
38	77	68	95	79	68	91
39	78	70	88	80	71	92
40	81	73	90	82	71	94

**Table 6:** Weekly diastolic blood pressure by postmenstrual age (mmhg) in infants with and without bronchopulmonary dysplasia

GA Mean	No BPD Diastolic BP (mmHg)			BPD Diastolic BP (mmHg)		
	5th	95th	Mean	5th	95th	
28	39	32	48	37	27	47
29	40	33	47	38	30	49
30	41	33	49	40	33	49
31	41	34	48	40	33	52
32	41	34	50	40	33	50
33	41	34	50	41	34	51
34	43	36	49	42	36	51
35	44	36	52	43	37	51
36	45	36	54	44	37	52
37	46	36	55	45	38	54
38	45	35	60	46	38	58
39	45	39	52	47	38	56
40	47	39	57	47	40	56

**Table 7:** Weekly mean blood pressure by postmenstrual age (mmhg) in infants with and without bronchopulmonary dysplasia

PMA (weeks	No BPD Mean BP (mmHg)			BPD Mean BP (mmHg)		
	GA Mean	5th	95th	Mean	5th	95th
28	48	41	57	46	35	58
29	49	42	57	48	37	60
30	50	43	58	49	41	60
31	50	43	58	50	43	63
32	51	44	59	50	44	60
33	51	44	59	51	43	62
34	53	45	59	52	45	61
35	54	45	62	53	46	62
36	56	47	64	55	47	63
37	56	45	64	56	48	66
38	56	47	72	57	48	69
39	56	49	63	58	50	68
40	58	52	68	59	50	69

Values are presented as mean and 5th–95th percentile ranges

Blood pressure measurements were obtained using non-invasive oscillometric methods and summarised by postmenstrual age

*BPD* Bronchopulmonary dysplasia, *BP* Blood pressure, *PMA* Postmenstrual age, *mmHg* Millimetres of mercury

**Fig. 2:**
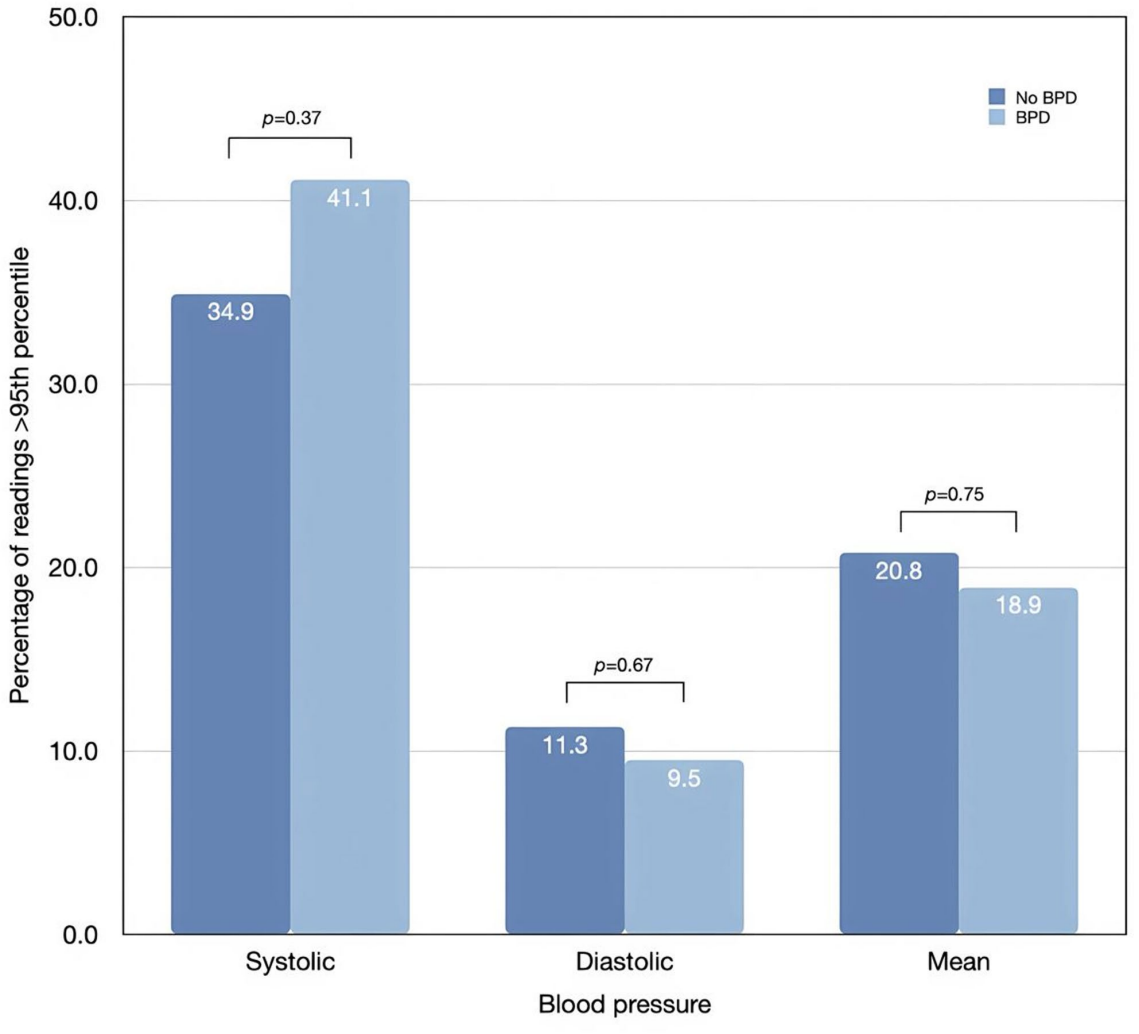
Hypertensive episodes in extremely preterm infants with and without bronchopulmonary dysplasia

Figure 2, a comparison between infants with and without BPD, showed no statistically significant difference in the proportion of hypertensive episodes demonstrated by elevated BP above the 95th percentile. Elevated systolic BP occurred in 41.1% of infants with BPD compared with 34.9% of those without BPD, while elevated diastolic and mean BP were observed in 9.5% vs. 11.3% and 18.9% vs. 20.8%, respectively, with no significant differences between groups.

**Fig. 3:**
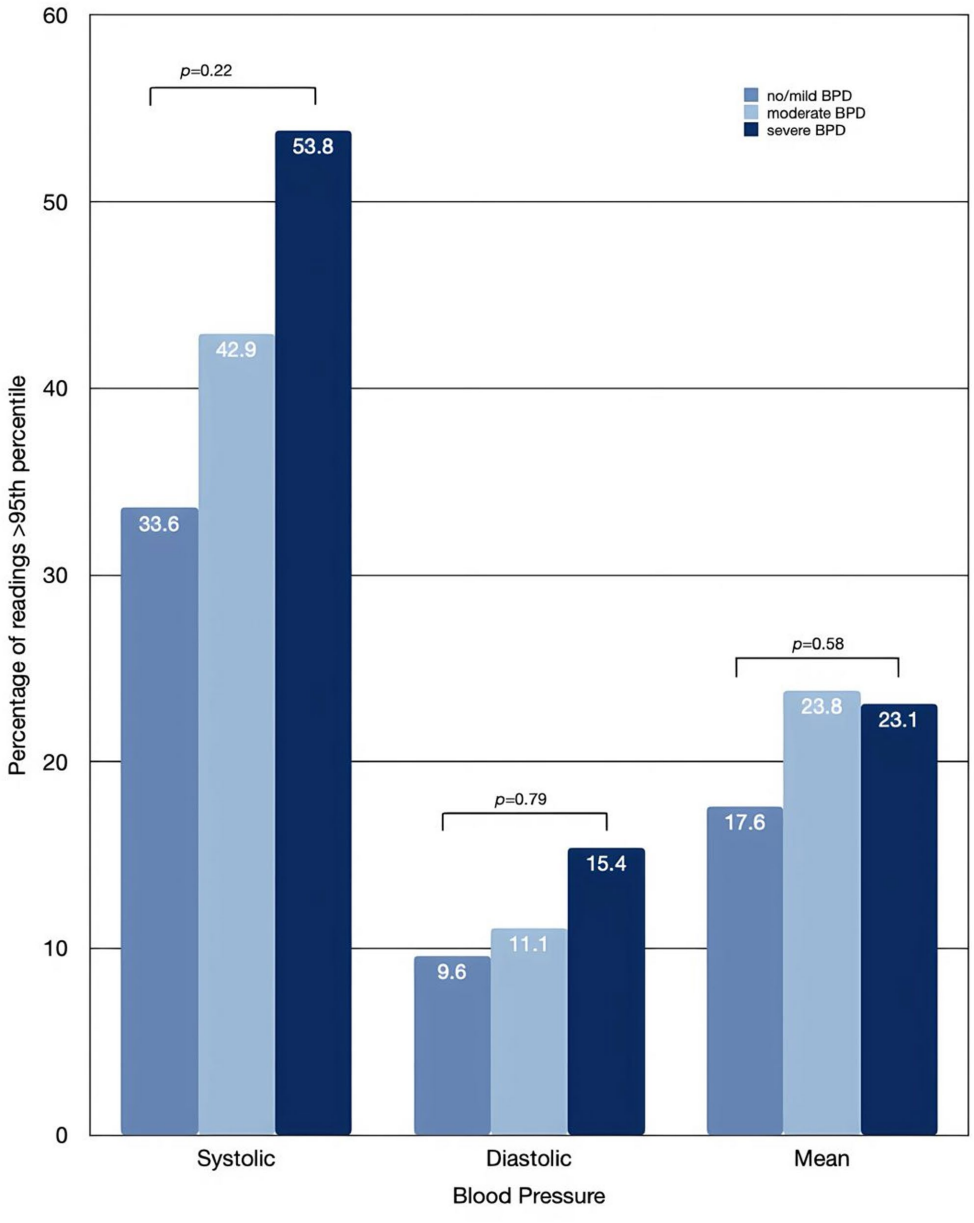
Comparison of hypertensive episodes across bronchopulmonary dysplasia severity groups

In Fig. 3, stratification by BPD severity demonstrated a stepwise increase in the proportion of infants with hypertensive episodes across severity categories. Elevated systolic BP was observed in 33.6% of infants with no or mild BPD, 42.9% with moderate BPD, and 53.8% with severe BPD. Similarly, elevated diastolic BP increased from 9.6% to 11.1% and 15.4%, and elevated mean BP from 17.6% to 23.8% and 23.1%, respectively. Although these trends suggest a dose-response relationship between BPD severity and blood pressure elevation, the differences did not reach statistical significance.

Multivariable logistic regression (Table 8) was performed to identify predictors of hypertensive BP episodes. Candidate variables entered the models included BW, GA, male sex, 1-minute Apgar score, surfactant administration, late-onset sepsis after two weeks of life, vasopressor exposure, PDA, diuretic therapy, BPD, duration of umbilical arterial catheterisation, and duration of mechanical ventilation.

For high systolic BP episodes, male sex (aOR 0.25, 95% CI 0.11–0.60, *p* = 0.002) and higher BW (aOR 0.996 per gram, 95% CI 0.993–1.000, *p* = 0.024) were independently associated with lower odds of systolic hypertension. For high diastolic BP episodes, male sex remained the only independent predictor (aOR 0.14, 95% CI 0.03–0.70, *p* = 0.017). For high mean BP episodes, male sex was again associated with lower odds of hypertensive episodes (aOR 0.24, 95% CI 0.09–0.68, *p* = 0.007), while GA showed a borderline association (aOR 0.64, 95% CI 0.40–1.01, *p* = 0.054). BPD and other clinical variables were not independently associated with hypertensive blood pressure episodes and were not retained in the final models.

**Table 8:** Multivariable logistic regression for hypertensive blood pressure episodes in extremely preterm infants

Outcome / Predictor	Adjusted Odds Ratio (95% CI)	*p* value
High systolic BP episodes		
Male sex	0.25 (0.11–0.60)	0.002
Birth weight (g)	0.996 (0.993–1.000)	0.024
High diastolic BP episodes		
Male sex	0.14 (0.03–0.70)	0.017
High mean BP episodes		
Male sex	0.24 (0.09–0.68)	0.007
Gestational age (weeks)	0.64 (0.40–1.01)	0.054

*BP* Blood pressure

## Discussion

This retrospective study is the largest cohort to characterise systemic BP patterns in growing EP infants with BPD. We found a biphasic distribution of readings; infants who later developed BPD had lower BP in the early postnatal period but higher BP at a later PMAs compared to infants without BPD. However, the differences in mean BP values were small (approximately 2–4 mmHg) and may not represent a clinically meaningful burden. Although a greater proportion of infants with BPD had BP readings within the hypertensive range (> 95th percentile), particularly for systolic BP (28.4% vs. 17.9%), the differences in BP values between groups across PMAs were generally small and did not reach statistical significance at most time points. Therefore, these longitudinal patterns should be interpreted cautiously and primarily as descriptive observations rather than evidence of a distinct BP phenotype associated with BPD.

Importantly, in multivariable regression analyses adjusting for clinical covariates, BPD was not independently associated with hypertensive BP episodes. Instead, male sex was associated with lower odds of hypertensive systolic, diastolic, and mean BP episodes, while higher BW was associated with fewer systolic hypertensive episodes, and GA showed a borderline association with mean BP episodes. These findings suggest that physiologic maturity and biological sex may have a stronger influence on BP variability in EP infants than BPD itself.

BP measurements in this study, stratified by PMA, are not directly comparable to previously published normative values derived from gestational age–based cohorts or studies using invasive or non-invasive methods [16–18], which reflect early postnatal physiology in relatively stable infants. In contrast, our measurements represent longitudinal, non-invasive assessments across evolving postnatal ages in EP infants with significant comorbidity. Differences in measurement technique, timing, and clinical status likely account for the observed discrepancies in BP patterns.

While previous studies have suggested that infants with BPD may have a higher prevalence of systemic hypertension, potentially reflecting alterations in cardiovascular development, and adding burden to the existing chronic lung disease – associated morbidities [9, 10], our analysis did not demonstrate an independent association between BPD and hypertensive BP episodes. However, the proportion of BP readings above the 95th percentile observed among infants with BPD in our cohort falls within the range previously reported in small cohorts of preterm infants born before 30 weeks’ gestation (12–43%) [9]. Given the lack of statistically significant differences between groups in our analysis, these findings should be interpreted with caution.

Our data also showed PMA-related BP patterns when comparing infants with and without BPD. The relatively lower BP in BPD infants during the first month of life in infants who later developed BPD may reflect the more critical condition and intensive care needs of these infants. In our cohort, BPD infants were of lower GAs and BWs and more often required invasive respiratory support from birth (e.g., higher rates of intubation and surfactant administration) [5]. These factors are known to correlate with early hemodynamic instability in EP neonates, suggesting overlapping pathways between initial cardiovascular compromise and later lung injury [19, 20]. BP comparisons were therefore performed using weekly PMA intervals rather than postnatal age to account for maturational effects.

The apparent reversal in BP trends toward term equivalent age in infants with BPD, albeit not statistically significant, may reflect evolving physiological changes associated with postnatal maturation and ongoing NICU exposures rather than a direct consequence of chronic lung disease alone. Alternatively, it may represent an early manifestation of developmental vascular remodelling in infants with BPD. Chronic lung disease may further contribute to these processes through systemic oxidative stress, elevated catecholamines, and inflammation, which can impair endothelial function and increase vascular reactivity [11]. Consistent with this concept, previous studies have reported that infants with BPD and systemic hypertension may also demonstrate evidence of renal stress or acute kidney injury [9]. In addition, common NICU therapies such as postnatal corticosteroids, diuretics, and caffeine may modestly influence BP patterns in EP infants [20–22]. These potential treatment-related influences highlight the complexity of interpreting BP patterns in this population.

The predominance of systolic over diastolic BP elevation observed in this cohort is biologically plausible. Systolic BP in preterm infants is more strongly influenced by cardiac output, arterial stiffness, and sympathetic tone, whereas diastolic pressure is largely governed by systemic vascular resistance [23–25]. Prior studies have reported that systemic hypertension in preterm infants, when present, tends to manifest predominantly as systolic elevation and may emerge around term-equivalent age [9, 26–29].

Male sex was associated with decreased odds of hypertensive systolic, diastolic, and mean BP episodes in the multivariable analysis. Sex differences in physiology that affect cardiovascular adaptation to early life may be important. Lower arterial BP has been reported in male preterm infants in the neonatal period, and this may be associated with differential vascular tone, hormone action, or cardiovascular transitional physiology [30, 31].

Markers of physiologic maturity were also of interest a priori. We found that higher BW was associated with fewer episodes of systolic hypertension, and GA was marginally associated with mean BP. Previous studies have shown that GA and BW are among the most important predictors of neonatal BP, indicating a gradual maturation of the cardiovascular system [25]. Taken together, our findings suggest that developmental maturity and biological sex may be more important determinants of BP variability in EP infants than the presence of BPD itself.

The results of our study should be interpreted considering the long-standing debates over the definitions and screening of neonatal hypertension. The definition of hypertension in preterm infants has not been universally agreed upon, as normal BP depends on GA, postnatal age, and measurement method [20]. In this study, we defined hypertension as a BP reading that is above the 95th percentile for PMA for our cohort distribution, which is consistent with commonly used approaches [20]. However, differences in reference curves and diagnostic thresholds across studies may contribute to variability in reported hypertension rates [9].

### Strengths and limitations

A key strength of this study is the longitudinal assessment of blood pressure in a well-defined cohort of EP infants. Weekly average BP measurements from 28 weeks PMA to NICU discharge allowed evaluation of temporal trends rather than reliance on single measurements. Consistent measurement methods, including standardised cuff sizing and non-invasive oscillometric monitoring within a single centre, improved comparability between groups. Additionally, infants were stratified by BPD severity to explore differences across disease subgroups.

Several limitations should be acknowledged. The retrospective design limited control of potential confounders, such as fluid balance and other clinical factors that may influence BP. The study was conducted at a single tertiary centre, which may limit generalizability. The moderate cohort size, particularly within BPD severity subgroups, may have reduced statistical power for some analyses. Oscillometric BP measurements may also overestimate systolic pressure and are sensitive to motion and cuff positioning, although averaging weekly readings helped mitigate this limitation. Finally, BP monitoring was limited to the NICU hospitalisation period, and longer-term follow-up is needed to better understand cardiovascular outcomes in this population.

## Conclusions

EP infants who developed BPD had different BP trajectories compared to those without BPD, characterised by early relative hypotension and slightly higher BP trends toward term-equivalent age (approximately 2–4 mmHg). Physiological age and sex-related cardiovascular differences, rather than BPD itself, are the predominant contributors to the observed BP variability.

Although differences in BP trajectories were modest and not consistently statistically significant, careful longitudinal BP monitoring during NICU hospitalisation and early follow-up after discharge may be warranted in EP infants. Further studies are needed to better understand the contributors to BP variability in this population and the potential long-term cardiovascular implications of early hemodynamic patterns.

## Data Availability

The de-identified data and all data supporting the findings of this study are available on reasonable request from the corresponding author. Data requests should be made to Dr Mohammad A. A. Bayoumi at moh.abdelwahab@hotmail.com.
